# Supplementary material for: A Gut Reaction to SIV and SHIV Infection: Lower Dysregulation of Mucosal T Cells during Acute Infection Is Associated with Greater Viral Suppression during cART
Source: Viruses. 2021 Aug 14;13(8):1609. doi: 10.3390/v13081609 (PMC8402906; doi:10.3390/v13081609)
Supplement: Supplementary file 1 [file viruses-13-01609-s001.zip › viruses-1291361-supplementary.pdf]

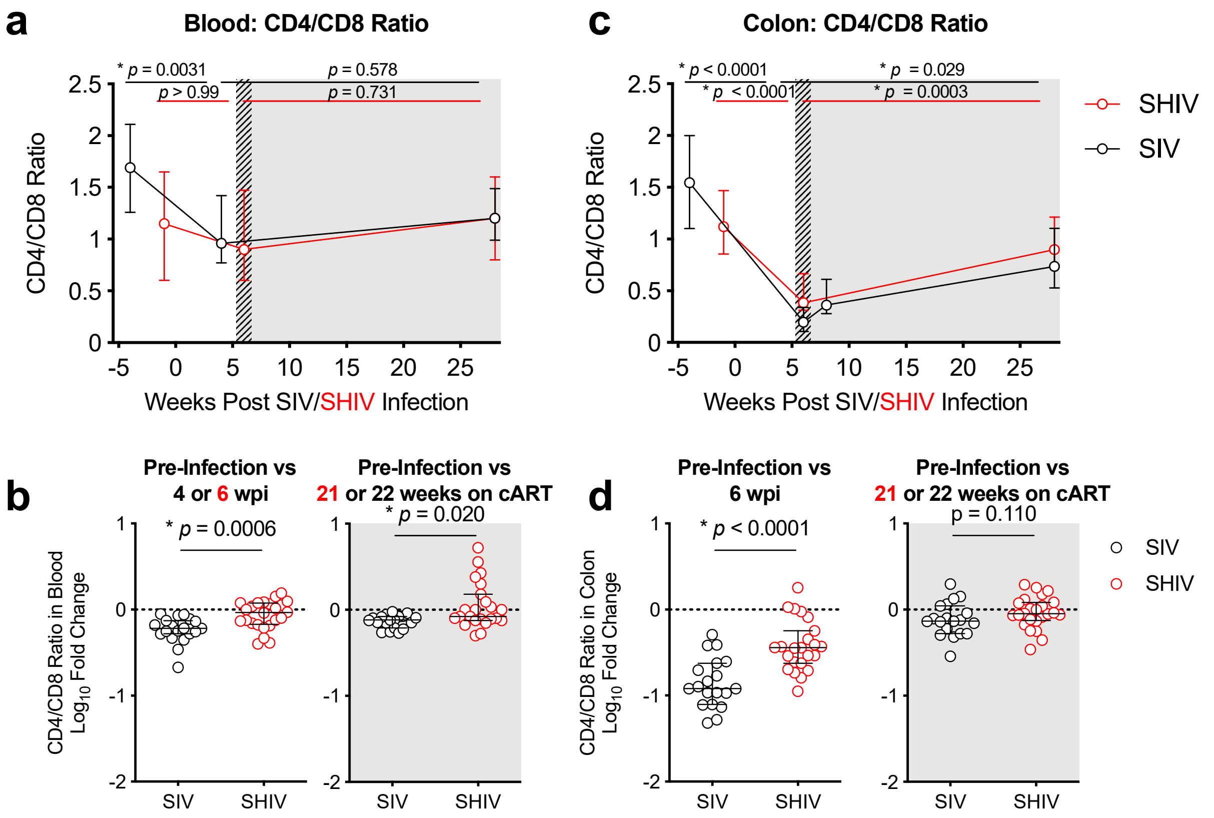

Supplemental Figure S1. Peripheral and mucosal CD4/CD8 ratios. The ratio of CD4/ CD8 in the blood (**a**) and colon (**c**) were quantified by dividing the frequencies of CD3+CD4+ by CD3+CD8+ as determined by flow cytometry. (**a, c**) Hashed and shaded regions indicate periods of cART treatment for SIV RMs and all animals, respectively. Lines indicate medians with interquartile ranges. Kruskal-Wallis test, adjusted p-values are given. (**b, d**) Log_{10}-fold change in CD4 T-cells between pre-infection and acute infection at 4 (SIV) or 6 (SHIV) weeks post-infection (wpi) or after 21 (SHIV) or 22 (SIV) weeks (>5 months) on cART in the blood (**b**) or colon (**d**) between SIV (black open circles) and SHIV (red open circles). Each dot represents an individual RM with medians and interquartile ranges indicated. The dotted line indicates no change (value of 1). *p*-values were determined using a Mann-Whitney test, with * *p*-values ≤ 0.05 considered significant.

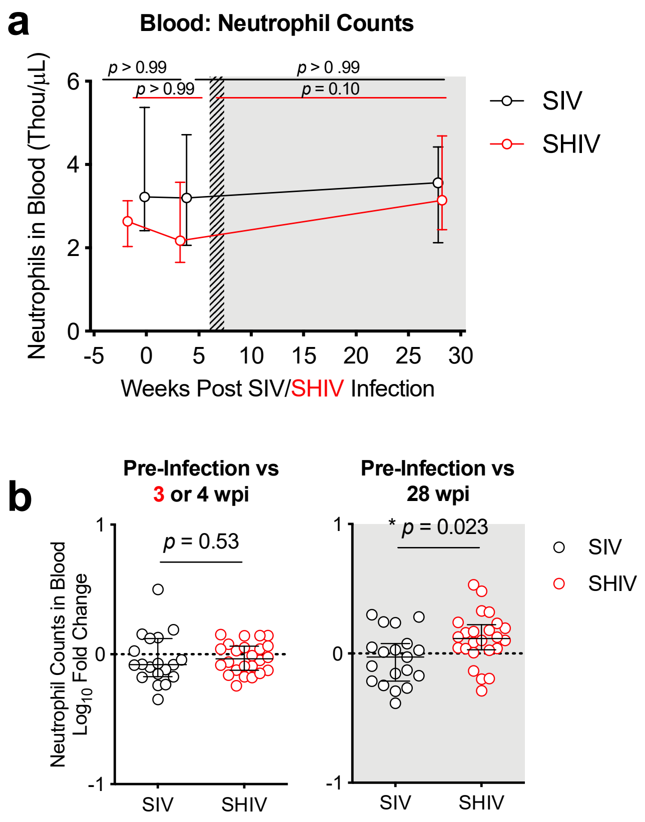

Supplemental Figure S2. Peripheral blood neutrophil counts. (**a**) The number of neutrophils per microliter of blood were quantified by complete blood count (CBC). Hashed and shaded regions indicate periods of cART treatment for SIV RMs and all animals, respectively. Lines indicate medians with interquartile ranges. Kruskal-Wallis test, adjusted p-values are given. (**b**) Log_{10}-fold change in neutrophil counts between pre-infection and acute infection at 3 (SHIV) or 4 (SIV) weeks post-infection (wpi) or after 21 (SHIV) or 22 (SIV) weeks (>5 months) on cART in the blood. Each dot represents an individual RM with medians and interquartile ranges indicated, SIV (black open circles) and SHIV (red open circles). The dotted line indicates no change (value of 1). *p*-values were determined using a Mann-Whitney test, with * *p*-values ≤ 0.05 considered significant.

Supplemental Table S1. Fold change in longitudinal immunological parameters evaluated in SIV- and SHIV-infected macaques.

	**Fold change from baseline (Mean ± Standard deviation)**		***p*-value**[a]
	SIVΔB670 N=19	SHIV-1157ipd3N4 N=24	SIV vs SHIV
**Blood CD3+CD4+ (Cells/∝L)**			
3-4 wpi	0.625 ± 0.175	1.24 ± 0.542	*< 0.0001*
28 wpi	0.784 ± 0.139	1.44 ± 0.642	*0.0001*
**Colon %CD4+**			
6 wpi	0.289 ± 0.152	0.643 ± 0.340	*< 0.0001*
28 wpi	0.818 ± 0.254	0.939 ± 0.308	0.14
**Blood CD4/CD8 Ratio**			
4-6 wpi	0.610 ± 0.178	1.38 ± 1.44	*0.0006*
28 wpi	0.742 ± 0.125	1.37 ± 1.14	*0.020*
**Colon CD4/CD8 Ratio**			
6 wpi	0.174 ± 0.128	0.482 ± 0.398	*< 0.0001*
28 wpi	0.846 ± 0.403	1.00 ± 0.412	0.110
**Blood Neutrophils (Thou/∝L)**			
3-4 wpi	1.02 ± 0.605	0.971 ± 0.261	*0.010*
28 wpi	1.02 ± 0.496	1.43 ± 0.688	0.11
**Colon %Treg**			
6 wpi	8.75 ± 14.4	1.51 ± 0.794	0.055
28 wpi	3.73 ± 2.39	1.58 ± 1.34	*0.0027*
**Colon %Th17**			
6 wpi	0.914 ± 1.68	1.38 ± 1.44	*0.010*
28 wpi	0.561 ± 0.459	0.875 ± 0.801	0.11
**Colon %Treg**			
6 wpi	8.75 ± 14.4	1.51 ± 0.794	0.055
28 wpi	3.73 ± 2.39	1.58 ± 1.34	*0.0027*
**Colon Th17/Treg Ratio**			
6 wpi	0.189 ± 0.201	1.95 ± 3.00	*< 0.0001*
28 wpi	0.343 ± 0.490	0.895 ± 1.11	*0.0027*
**Colon Th17 Polyfunc.**[b]			
6 wpi	0.996 ± 1.71	1.02 ± 0.909	0.12
28 wpi	0.494 ± 0.385	2.93 ± 5.70	*0.012*
**Colon %Ki67+/CD4+**			
6 wpi	4.29 ± 3.96	2.30 ± 1.50	0.10
28 wpi	1.85 ± 1.21	1.11 ± 1.34	*0.007*
**Colon %Ki67+/CD8+**			
6 wpi	3.19 ± 3.76	2.37 ± 1.54	> 0.99
28 wpi	1.26 ± 1.36	0.800 ± 0.948	*0.042*

[a] Mann-Whitney test was used to generate *p*-values; $p<0.05$ is considered significant and indicated by italicized text.
[b] Th17 Polyfunc. (%): Polyfunctional was defined as the percentage of cells expressing 3 cytokines: IL-17 and ≥ 2 cytokines (IL-22, IFNγ, TNFα, or IL-2).
Abbreviations: polyfunc. (polyfunctional)
